# Overlapping *Podospora anserina* Transcriptional Responses to Bacterial and Fungal Non Self Indicate a Multilayered Innate Immune Response

**DOI:** 10.3389/fmicb.2016.00471

**Published:** 2016-04-19

**Authors:** Marina Lamacchia, Witold Dyrka, Annick Breton, Sven J. Saupe, Mathieu Paoletti

**Affiliations:** ^1^Institut de Biologie et Génétique Cellulaire, UMR 5095, Centre National de la Recherche Scientifique et Université de BordeauxBordeaux, France; ^2^Equipe MAGNOME, INRIA, Université de Bordeaux, Centre National de la Recherche ScientifiqueTalence, France; ^3^Department of Biomedical Engineering, Faculty of Fundamental Problems of Technology, Wroclaw University of TechnologyWroclaw, Poland

**Keywords:** bacterial fungal interaction, vegetative incompatibility, *Podospora anserina*, *Serratia marcescens*, *Serratia fonticola*, transcriptional response

## Abstract

Recognition and response to non self is essential to development and survival of all organisms. It can occur between individuals of the same species or between different organisms. Fungi are established models for conspecific non self recognition in the form of vegetative incompatibility (VI), a genetically controlled process initiating a programmed cell death (PCD) leading to the rejection of a fusion cell between genetically different isolates of the same species. In *Podospora anserina* VI is controlled by members of the *hnwd* gene family encoding for proteins analogous to NOD Like Receptors (NLR) immune receptors in eukaryotes. It was hypothesized that the *hnwd* controlled VI reaction was derived from the fungal innate immune response. Here we analyze the *P. anserina* transcriptional responses to two bacterial species, *Serratia fonticola* to which *P. anserina* survives and *S. marcescens* to which *P. anserina* succumbs, and compare these to the transcriptional response induced under VI conditions. Transcriptional responses to both bacteria largely overlap, however the number of genes regulated and magnitude of regulation is more important when *P. anserina* survives. Transcriptional responses to bacteria also overlap with the VI reaction for both up or down regulated gene sets. Genes up regulated tend to be clustered in the genome, and display limited phylogenetic distribution. In all three responses we observed genes related to autophagy to be up-regulated. Autophagy contributes to the fungal survival in all three conditions. Genes encoding for secondary metabolites and histidine kinase signaling are also up regulated in all three conditions. Transcriptional responses also display differences. Genes involved in response to oxidative stress, or encoding small secreted proteins are essentially expressed in response to bacteria, while genes encoding NLR proteins are expressed during VI. Most functions encoded in response to bacteria favor survival of the fungus while most functions up regulated during VI would lead to cell death. These differences are discussed in the frame of a multilayered response to non self in fungi.

## Introduction

All living organisms have developed mechanisms to perceive and respond to non self. Recognition can be conspecific, between individuals of the same species, or heterospecific in the context of host/pathogen or host/symbiont interactions. Fungi display a whole range of beneficial or detrimental heterospecific interactions, with plants, animals, other fungi, bacteria, or viruses. In particular, Bacterial Fungal Interactions (BFI) have been extensively studied in different contexts (Frey-Klett et al., 2011), including situations where fungi are targeted by pathogenic bacteria (Leveau and Preston, 2008). However, fungal immune receptors remain largely uncharacterized and the fungal reaction to bacterial pathogens is only beginning to be investigated at the molecular and transcriptional level. Large scale transcriptomic analysis of BFI include pathogenic interactions between *Aspergillus niger* and *Collimonas fungivorans* (Mela et al., 2011) or *Rhizoctonia solani* with *Serratia* species (Gkarmiri et al., 2015), cooperative interaction between *A. niger* and *Bacillus subtillis* (Benoit et al., 2015) or comparative analysis of different behaviors between *Laccaria bicolor* and three different bacterial species (Deveau et al., 2015). Fungi also display a con-specific non self recognition mechanism called vegetative incompatibility (VI) which is genetically controlled and leads to the rejection of conspecific non self generated by anastomosis between genetically different isolates of the same species. Co-expression in the same cytoplasm of incompatible alleles of so-called *het* genes initiates the VI reaction that culminates with a programmed cell death (PCD) reaction of the fusion cell, thereby maintaining different isolates separated (Glass et al., 2000; Saupe, 2000). VI has been shown to be selectively advantageous in some circumstances as it can restrict resource plundering (Debets and Griffiths, 1998), horizontal propagation of cytoplasmic viruses (Choi et al., 2011), or deleterious plasmid (Debets et al., 2012). *het* genes have been characterized in three fungal species, *Podospora anserina, Neurospora crassa*, and *Cryphonectria parasitica* (Saupe, 2000; Choi et al., 2011). Although not related in sequences, these genes always display a high degree of allelic polymorphism.

STAND proteins are tripartite signal transduction proteins (Leipe et al., 2004), and in plants and animals the vast majority of STAND proteins are innate immune receptors, including NOD like receptors in animals and NB-LRR resistance proteins in plants (Rairdan and Moffett, 2007). Plant innate immune response is a multilayered process. A first line of defense called Pattern Triggers Immunity (PTI) is initiated upon recognition of conserved pathogen molecular markers. Adapted pathogen then develop effecters whose functions are to alter components of the innate immune response, and NB-LRR receptors in turn recognize these effecters to initiate the Effecter Triggered Immunity (ETI). Detection of pathogens effecters occurs either by direct interaction with the NB-LRR receptors, but most frequently NB-LRR receptors sense alterations of host proteins as a consequence of the effecters action in a model known as guardian guardee (Jones and Dangl, 2006) recently reviewed (Khan et al., 2016). This model has also been described for animal NOD like receptors (Ferrand and Ferrero, 2013). Fungal genomes do not encode for NB-LRR proteins (Soanes and Talbot, 2010; Dyrka et al., 2014) but instead encode for a great diversity of STAND proteins (Dyrka et al., 2014). Some of the protein domains constituting fungal STAND proteins display phylogenetic relationship to domains involved in immunity in plant and mammals, including the central nucleotide binding NACHT domain (Koonin and Aravind, 2000), or the N terminal HET domain related to plant and animal TIR domain (Dyrka et al., 2014). Interestingly some *het* genes in *P. anserina* and *C. parasitica* encode for proteins of the STAND family (Saupe et al., 1995; Choi et al., 2011). In *P. anserina het-d* and *het-e*, members of the *hnwd* gene family encoding STAND proteins (Paoletti et al., 2007), form non allelic incompatibility systems with *het-c* encoding a glycolipid transfer protein (Saupe et al., 1994). *het-c* and *hnwd* genes are subjected to positive diversifying selection (Chevanne et al., 2010; Bastiaans et al., 2014), which can be best explained in the context of and evolutionary arms race with pathogens. In addition, by many aspects VI is analogous to hybrid necrosis in plants, a plant auto-immune disease associated with NR-LRR immune receptors (Bomblies et al., 2007). These observations lead to the proposition that VI in *P. anserina* was derived from the *P. anserina* innate immune system (Paoletti and Saupe, 2009). In this hypothesis HET-c protein would be under the surveillance of HNWD innate immune receptors in the frame of the guardian guardee model (Jones and Dangl, 2006). Accordingly HET-c protein would be targeted by pathogen effecters and HNWD receptors recognize these alterations. Pathogen driven fast evolution of *hnwd* (Paoletti et al., 2007) and *het-c* (Bastiaans et al., 2014) genes would occasionally generate incompatible combinations of alleles maintained for VI. In a similar fashion *acd11*, an *A. thaliana* homolog of *het-c*, encodes a protein proposed to be under the surveillances of the NB-LRR receptor LAZ5 (Palma et al., 2010). This model has several implications, including the fact that HET-c contributes to the defense response against non self, and also that immune response to pathogens and VI reactions induce similar responses. To assess this model we have selected two related bacterial species, *Serratia marcescens* and *S. fonticola*, that trigger a reaction in *P. anserina* WT isolates that is altered in a Δ*het-c* knock out mutant. Briefly Δ*het-c* mutants appear more sensitive than wt *P. anserina* to both bacterial species, the full description of the selection process will be presented elsewhere. Here we describe the interactions of WT *P. anserina* to the two *Serratia* species that result either in death or survival of the fungus. We also report the *P. anserina* transcriptional responses to these bacteria as analyzed by a RNA-seq approach. We compare these responses to the STAND triggered VI reaction. While the transcriptional responses to bacteria significantly overlap with the VI reaction in different aspects they also display specificities. These results are discussed in the frame of a multilayered fungal response to heterospecific non self.

## Materials and methods

### Strains and culture conditions

The WT *P. anserina* strain Cs is almost isogenic to the strain whose genome was sequenced (Espagne et al., 2008), except for the *het-s* VI locus. Pa-ATG8-GFP, IDI-GFP, and IDI2-GFP as well as confocal microscopy methods were described previously (Dementhon et al., 2003; Pinan-Lucarré et al., 2003). *S. fonticola* was isolated from compost and identified by PCR amplification and sequencing of 16S ribosomal DNA encoding region. *S. marcescens* DB11 strain was a kind gift from D. Ferrandon's lab.

For confrontation assays six spots of 10 μl of a bacterial suspension from a fresh pre culture (OD600 nm = 1) were laid on top of a corn meal agar plate, and six plugs of fungal mycelium were laid about 1 cm from the bacterial spots. The cultures were left to grow at 26°C in the dark. For transfer assays fungal cultures were set up on cellophane stripes on corn meal agar, and bacterial cultures were set up independently as three rows of 10 μl spots (OD600 nm = 1) on the same medium at 26°C in the dark. After 48 h incubation, cellophane stripes were transferred onto bacteria seeded plates between rows of bacterial spots.

### RNA extraction

After 2 or 6 h incubation, the mycelium ware scraped off the cellophane and freeze dried for 48 h. RNA was extracted with Qiagen RNA plant minikit and DNAse treated. As control RNA was extracted from fungal mycelium transferred to bacteria free plates. For the VI condition a self-incompatible thermosensitive strain was grown on corn meal agar and treated as described (Bidard et al., 2013). All experiments were duplicated.

### RNA-Seq and differential expression analysis

cDNA library construction and sequencing, mapping to *P. anserina* annotated genes and differential expression analysis were performed by Beckman Coulter Genomics using the Trinity pipeline. We selected genes with a log_2_FC > 2 and *p* < 0.01 for further analysis.

### Sequence analysis

Gene Ontology (GO) assignation (https://www.blast2go.com/), Pfam annotation (http://pfam.xfam.org/), and promoter sequence analysis (http://genie.dartmouth.edu/scope/) were performed with default cut off values. Two tails Fisher's or Chi2 statistical tests were conducted when required. Orthologous genes were identified as reciprocal best hits.

### Versatility of *P. anserina* genes

Versatility of *P. anserina* genes assignment depends on the number of Blastp hit results against NCBI nr database obtained at the genus level by their translation products. For each *P. anserina* protein we counted the number of Blastp hit (cut off: *p* < ^*1e-*5^). Genes whose products result in a single hit (itself) are categorized as orphans, while genes having an ortholog in *S. cerevisiae* are considered as part of the fungal core genome. The remaining genes are arranged in versatility bins numbered one to 10 of about 670 genes each, depending on the increasing number of hits, so that genes in the lower numbered categories produced the fewer hits. Genes were then assigned a versatility index, set to 0 for orphans, 11 for genes belonging to the fungal core genome, and the bin number for the remaining genes. A sliding window analysis of the distribution of gene versatility along chromosomes was performed with a window size of 100 genes. Regions of high versatility level correspond to contiguous genes with versatility value below average versatility minus standard deviation for the whole chromosome.

### Confocal imaging and cell death quantification

Light and fluorescent observations were performed on a Leica DRMXA confoncal microscope. Cell death was measured as described previously after staining with the Evans blue vital dye (Pinan-Lucarré et al., 2005), basically comparing the length of dead to living fungal hyphae.

### ROS detection

The fungus was soaked in a solution of nitrotetrazolium blue chloride (NBT, 250 μg/ml) for 20 min, washed extensively with water before observing under the microscope. NBT forms a blueish precipitate in presence of ROS.

## Results and discussion

### Phenotypic characterization of *P. anserina* response to *Serratia* species

**Figure 1:**
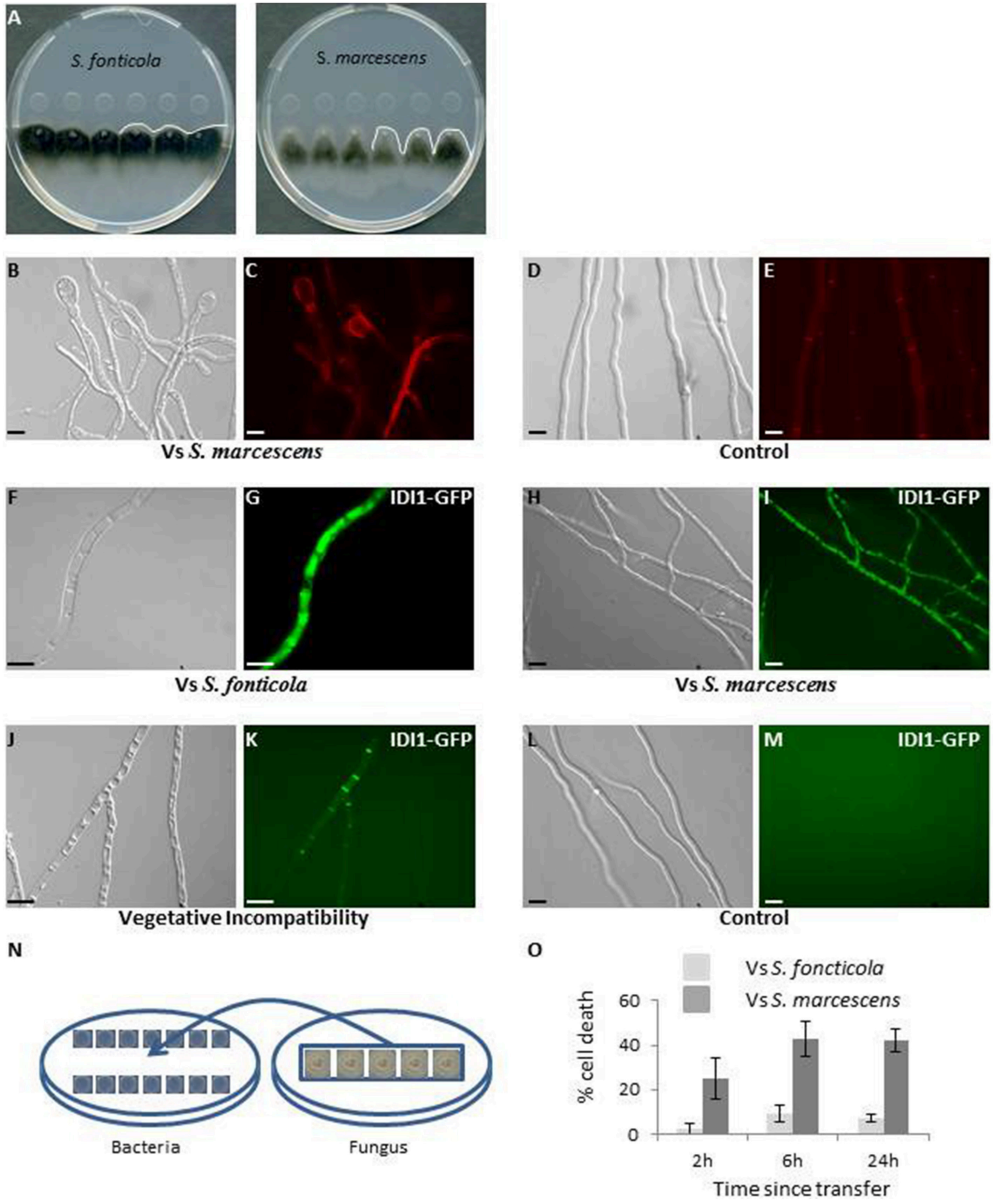
**Phenotypic characterization of the interaction between *P. anserina* and *S. fonticola* or *S. marcescens*. (A)** Confrontation assay against *S. fonticola* and *S. marcescens*. Fungal growth toward the bacterial colonies stops before contact is made. Fungal colony edge appears linear in confrontation to *S. fonticola* and is altered in confrontation to *S. marcescens*. The white line delineates the fungal colony edge. **(B)** Fungal cell morphology appears altered on the side of the bacterial colony, especially at apices, with cells swelling in confrontation with both bacterial species, here *S. marcescens*. **(C)** Cell death is observed by Evans Blue staining in confrontation with both bacteria. **(D)** Cell morphology and **(E)** Evans Blue staining in absence of bacteria. **(F–K)** Large vacuoles **(F,H,J)** and expression of IDI1-GFP **(G,I,K)** are observed in presences *S. fonticola* **(F,G)** or *S. marcescens* **(H,I)**, or during the VI reaction **(J,K)** but not in cells growing away from the bacterial colony **(L,M)**. Scale bars = 10 μm. The same induction of expression and localization is observed for Pa-GFP-ATG8 and IDI2-GFP proteins (not shown). **(N)** In the transfer assay, bacteria and *P. anserina* (on cellophane stripes) are grown for 48 h on separate plates, and the fungus is then transferred onto the bacteria seeded plates, setting the initial time point of the reaction. **(O)** Estimation of the fungal cell death level after transfer to *S. fonticola* or *S. marcescens* seeded plates.

In a confrontation assay against *S. marcescens* or *S. fonticola, P. anserina* grew normally away from the bacterial colony, but growth toward the bacterial colony soon stopped before contact was made. Where the edge of the fungal colony appears almost linear in confrontation to *S. fonticola*, it appears altered in confrontation to *S. marcescens* (Figure 1A). After 2–3 days growth resumed in the confrontation to *S. fonticola* and the fungal colony eventually covered the bacterial colony. In the confrontation to *S. marcescens* growth did not resume. Fungal growth arrest in confrontation with *Serratia* species has already been reported (Li et al., 2015). Fungal cell morphology was altered in confrontation to both bacteria, with an intense vacuolization, apical cell swelling, and occasional cell death (Figures 1B–E). These phenotypes appeared more pronounced in confrontation to *S. marcescens*. We also observed the induction of autophagy as indicated by the vacuolar localization of Pa-GFP-ATG8, along with the expression of IDI1-GFP and IDI-2-GFP, two small secreted proteins induced during VI and believed to act as defensins (Figures 1F–K; Bourges et al., 1998; Dementhon et al., 2003). During VI IDI1-GFP and IDI2-GFP are localized to the membrane, while in response to bacteria they appear vacuolar, which could be a consequence of bacterial toxins altering the *P. anserina* secretory pathway (Guichard et al., 2014). These phenotypes decrease in intensity as observations are made further away from the colony edge, and are not observed in fungal cells growing opposite from the bacterial colony (Figures 1L,M).

We also developed a transfer assay where *P. anserina* mycelium is grown on cellophane stripes on a bacteria free medium before transfer onto a bacteria seeded medium (Figure 1N). This transfer assay results in induction of a response in the entire mycelium as revealed by monitoring induction of IDI1-GFP, IDI2-GFP, and autophagy. This assay also allows for the timing of the response. We estimated *P. anserina* cell death level over time (Pinan-Lucarré et al., 2005). Transfer to *S. fonticola* seeded plates resulted in a low (<10%) and transient level of cell death while transfer to *S. marcescens* seeded plates lead rapidly to a considerable level of cell death (>40%; Figure 1O). Taken together these results indicated that *P. anserina* was resistant to *S. fonticola* but sensitive to *S. marcescens* in our conditions.

### High numbers of genes up and down regulated

Using a RNA-seq approach we analyzed *P. anserina* transcriptional response 2 and 6 h after transfer to *S. marcescens* or *S. fonticola* seeded plates compared to a control response after transfer to bacteria free plates. We also sequenced pooled RNA extracted 1 and 3 h after induction of VI, conditions largely covering the VI transcriptional response as previously described (Bidard et al., 2013). Number of genes up and down regulated by at least a factor 2, and maximum fold changes are reported in Table 1. Number of up regulated genes range from 1091 to 1913 (10–18% of the genome), while number of genes down regulated genes vary from 781 to 1923 (7–18% of the genome).We pooled expression data obtained at different times of exposure to bacteria to generate the set of unique genes regulated in presence of *S. fonticola* (VsSf) or *S. marcescens* (VsSm) (Table 1). The number of genes up or down regulated are similar in response to bacteria or during VI.

**Table 1:** ***P. anserina* genes differentially expressed in response to non self**.

	**Vs** ***S. fonticola***			**Vs** ***S. marcescens***			**Incompatibility**
	**2 h**	**6 h**	**VsSf**	**2 h**	**6 h**	**VsSm**	**1 and 3 h**
Up	1232	1325	1847	1091	1289	1668	1913
Max log_2_Fc	10.2	8.8	10.2	9.2	8.5	9.2	11.5
Down	838	1605	1882	781	1458	1615	1923
Max log_2_Fc	−9.5	−9.9	−9.9	−10.1	−8.4	−10.1	−9.5

*Number of genes up and down regulated after transfer 2 or 6 h to S. fonticola or S. marcescens seeded plates, or during the vegetative incompatibility reaction are reported. VsSf and VsSm genes sets correspond to the total number of genes differentially regulated in presence of S. fonticola or S. marcescens at least one of the two periods of incubation, respectively*.

### Responses to bacteria overlap

**Figure 2:**
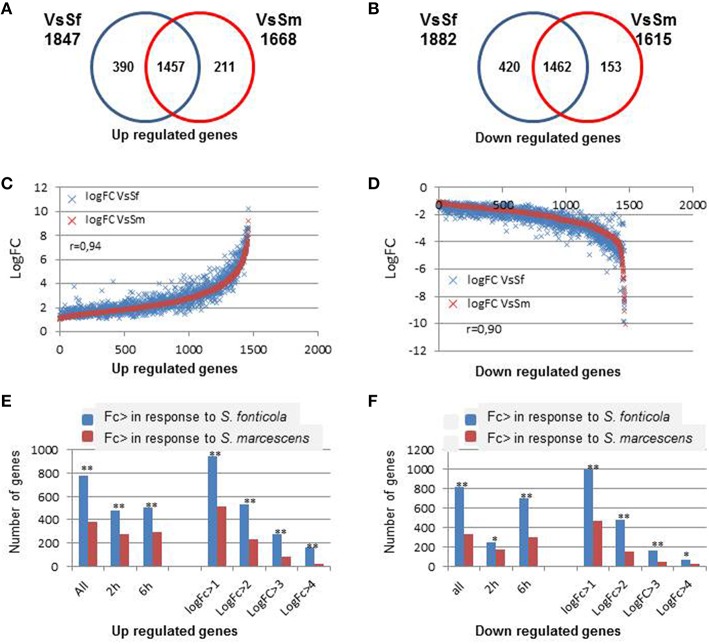
**Comparison of the differentially expressed genes in response to bacteria. (A,B)** Venn diagram showing *P. anserina* genes up or down regulated in response to *S. fonticola* or *S. marcescens*. **(C,D)** Correlation of the fold change level in response to both bacteria. For each gene expressed in both conditions, LogFc levels in response to both bacteria are reported. Genes are ordered along the X-axis according to increasing LogFc values **(C)** in response to *S. marcescens* for genes up regulated and decreasing LogFc values **(D)** in response to *S. marcescens* for genes down regulated. **(E,F)** Magnitude of transcriptional regulation is more important in response to *S. fonticola* than in response to *S. marcescens*. Histograms represent the number of genes that are more up **(E)** or down **(F)** regulated in response to *S. fonticola* or *S. marcescens* in the VsSf or VsSm data sets, at each time point, or for different LogFc values. Fisher tests were conducted to compare number the number of genes with a greater level of regulation in response to both bacteria (^**^p < 0.001, ^*^p < 0.05).

We first compared *P. anserina* transcriptional responses to both bacteria. As indicated in Figure 2, both responses largely overlap (Fisher test, *p* = 0), and the response to *S. marcescens* is almost entirely included in the response to *S. fonticola*. Only 12 and 9.5% of the genes up or down regulated in the VsSm set were not in the VsSf set, while 21 and 22% of the VsSf set were not in the VsSm set. This observation remains true when looking at regulated genes after 2 or 6 h of exposure to the bacteria, or when looking at expressed genes with different LogFc levels. Three hundred and ninety and 211 are up regulated specifically in response to *S. fonticola* or *S. marcescens*, while 420 and 153 genes are specifically down regulated in response to these two bacteria, respectively. When focusing on genes regulated in both conditions we observed a good correlation in the response to both bacteria (Figure 2), meaning that globally in response to both bacteria gene expression regulation follows a similar pattern. However, we observed that generally the magnitude of the differential expression, whether for up or down regulation, is more important in response to *S. fonticola* than in response to *S. marcescens* (Figure 2). In other words globally genes are more up or down regulated in response to *S. fonticola* than to *S. marcescens*. Again, this remains true for different times of exposure or when considering different logFc level.

Overall, the response to *S. fonticola* includes more genes with a greater level of differential expression. Whether the difference comes from a better ability of *P. anserina* to detect and respond to *S. fonticola* than to *S. marcescens*, or from a better ability of *S. marcescens* to subdue *P. anserina* response than *S. fonticola* remains to be investigated.

### Transcriptional responses to bacteria overlap with vegetative incompatibility

**Figure 3:**
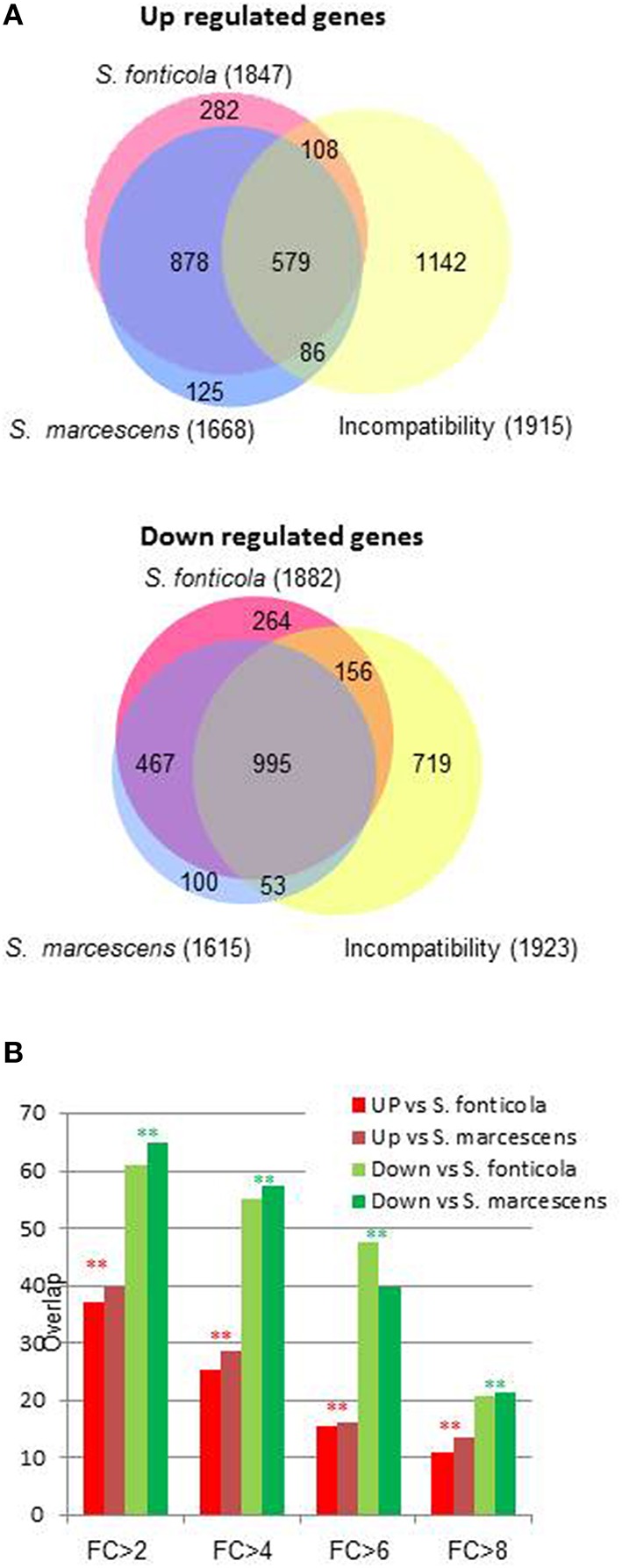
**Overlap between transcriptional response to bacteria and vegetative incompatibility. (A)** Area proportional Venn diagram showing *P. anserina* genes up or down regulated after transfer to *S. fonticola* or *S. marcescens* seeded plates, and during the vegetative incompatibility reaction. Number in brackets represent the total number for each gene set with a FC > 2 and *p* < 0.01, values within the graph represent number of genes for each intersect. **(B)** Histogram representing the percentage of genes up or down regulated by the presence of *S. fonticola* or *S. marcescens* that are also up or down regulated during the VI reaction for different fold change values. In all cases the overlap between response to bacteria and VI response is greater than expected by chance (^**^p < 0.001, ^*^p < 0.01).

We next observed that transcriptional response to bacteria also significantly overlap with the VI reaction (Chi2, *p* = 0; Figure 3). The overlap is significantly more important for genes down regulated than for genes up regulated (Chi2, *p* = 0). As the fold change increases, the overlaps between responses to bacteria and VI decrease. Only six and 15 genes are common to the 100 most up or down regulated genes in all three conditions. Transcriptional responses to VI and to bacteria thus share a common signature for genes with a low level of regulation while genes the most regulated in response to bacteria are different from the genes most regulated during VI.

### Genes up regulated in response to non self tend to be lineage specific

**Figure 4:**
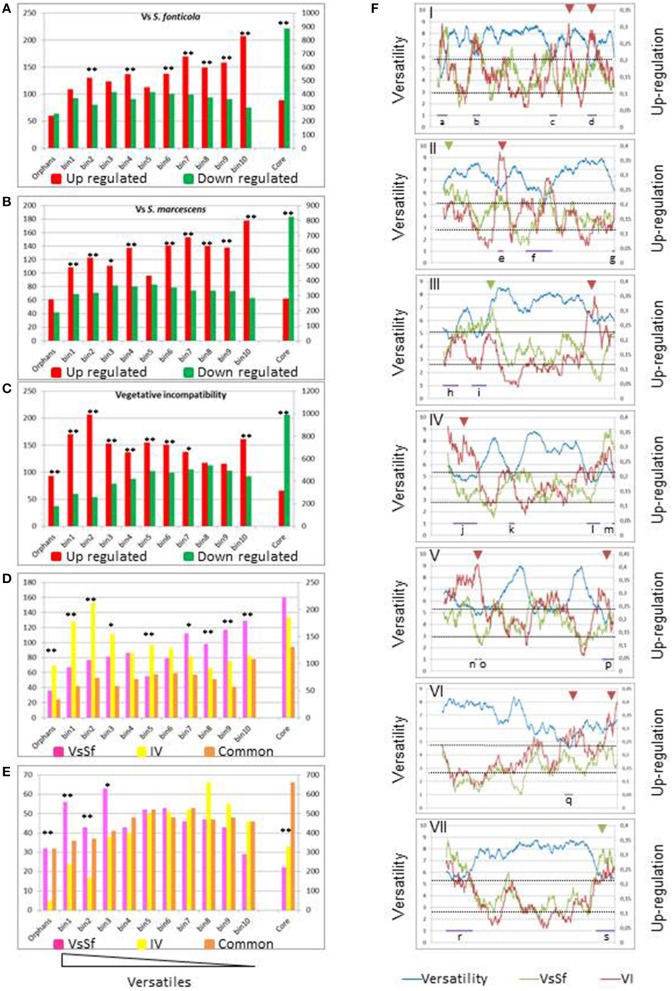
**Versatility of differentially expressed genes. (A–C)** The histograms represent the number of differentially expressed genes in response to non self for each versatility category. The left scale is relevant for number for orphan or versatile genes, the right scale for core genome genes. Fisher's tests were conducted to compare number of up and down regulated genes in each category (^*^p < 0.05, ^**^p < 0.001). **(D,E)** Number of genes up or down regulated during VI only, in response to *S. fonticola* only or in both conditions. Fisher's test were conducted to compare the number of genes up or down regulated specifically in either condition (^*^p < 0.05, ^**^p < 0.001). **(F)** Distribution of the versatility level (blue line) or up regulated genes in response to *S. fonticola* (Red line) or during VI (green line) along each *P. anserina* chromosomes. Each of the seven *P. anserina* chromosomes was analyzed by a sliding window analysis (window size of 100 genes). For each 100 genes window, versatility is expressed as an average value (left axis), while fraction of up regulated genes is referred to the right axis. The horizontal axis represents the chromosome. Purple bars (lettered a–s) indicate regions of high density of versatile genes; arrowheads indicate regions of high density of expression specific to a given condition (green for VsSf, red for VI).

Wapinski and co-authors introduced the term versatility to describe the frequency at which genes can be gained or lost during the course of evolution, and they found that genes encoding for adaptive functions are more versatile than genes encoding for essential functions (Wapinski et al., 2007). In previous studies of VI (Hutchison et al., 2009; Bidard et al., 2013), showed that up regulated gene sets included an excess of orphan genes. We analyzed versatility of genes regulated in response to non self. We approached versatility of *P. anserina* annotated cds by counting the number of blastp hits (cut off *e*-value < 1^*e-*5^) of their predicted products at the genus level in fungal genomes. We defined the fungal core genome as the genes having an ortholog in the distantly related ascomycete *S. cerevisiae* (3297 genes described in Bidard et al., 2013), orphans as having a blastp hit only in *P. anserina* (640 genes) and ranked the remaining genes (6698 sequences) in 10 versatility bins of approximately 670 sequences according to an increasing number of hits, so that smallest numbered bins contain sequences resulting in the less blastp hits (all bin compositions are presented in Additional file 1). Genes belonging to the versatile bins or orphan categories are significantly more up than down regulated [1493 up/995 down (*p* = 6.^2*e-*28^) for VsSf, 1386 up/790 down (*p* = 0) for VsSm, 1597 up/932 down (*p* = 0) for VI]. In contrast the core genome is more down than up regulated [354 up/886 down (*p* = 0) for VsSf, 282 up/825 down (*p* = 0) for VsSm, 317 up/991 down (*p* = 0) for VI]. We then analyzed the distribution of differentially expressed genes in these categories for the three responses we analyze (Figures 4A–C). In all three responses genes belonging to orphan or versatile categories are more up than down regulated. However, repartition of differentially expressed genes in versatility bins differ between VI and response to bacteria as exemplified in the comparison between VsSf and VI gene sets (Figures 4D,E). Genes up regulated during VI are more represented in the most versatile bins, while genes up regulated in response to the bacteria are more represented in the less versatile bins. Genes expressed in both conditions are equally represented in all bins. Inversely, genes down regulated in response to bacteria are more represented in the most versatile bins. The situation is identical when comparing the reaction to *S. marcescens* and VI (not shown).

### Versatile genes are clustered

We next analyzed distribution of versatile and conserved genes on chromosomes along with their expression. Genes were assigned a versatility value (0 for orphans, 11 for core genome genes, and the versatility bin numbers for the rest) and we performed a sliding window analysis of the versatility level of genes along the chromosomes. As expected versatile genes are not evenly distributed (Fedorova et al., 2008; Klosterman et al., 2011) and we identified a total of 19 regions of high density of versatile genes distributed between all chromosomes, eight of which appear to correspond to telomeric/subtelomeric regions (Figure 4F). We also identified chromosomal regions with a high density of genes up regulated in response to non self. Thirteen out of 19 (lettered a–i) highly versatile regions coincide with regions of high up-regulation density in at least one condition. Note that inversely regions of highly conserved genes are essentially down regulated (Additional file 2). There is thus a clear correlation between versatility gene distribution and expression landscape in response to non self. We also identified regions of high expression level specific either to the response to bacteria or to VI (Figure 4F). Clustering of versatile genes could facilitate their coordinated epigenetic transcriptional regulation, as proposed for the regulation of effector encoding genes in the plant pathogen *Leptospheria maculans* (Soyer et al., 2014).

Overall, the core genome is essentially down regulated while less conserved genes are up regulated. This is particularly true for the VI reaction that includes expression of an additional set of highly versatile genes. Upregulated versatile genes tend to cluster in specific regions of the genome. Expression of versatile genes in response to non self suggest an adaptive nature of the responses to non self (Wapinski et al., 2007).

### Functional annotation of differentially expressed genes

We analyzed Gene Ontology (GO) terms and Pfam-A protein domains associated with differentially expressed genes. As expected, annotation level is correlated with the versatility of the genes (Additional file 3), and thus the proportion of annotated up regulated genes is inferior to that of down regulated genes for both GO terms and Pfam-A domains (Additional file 3).

**Figure 5:**
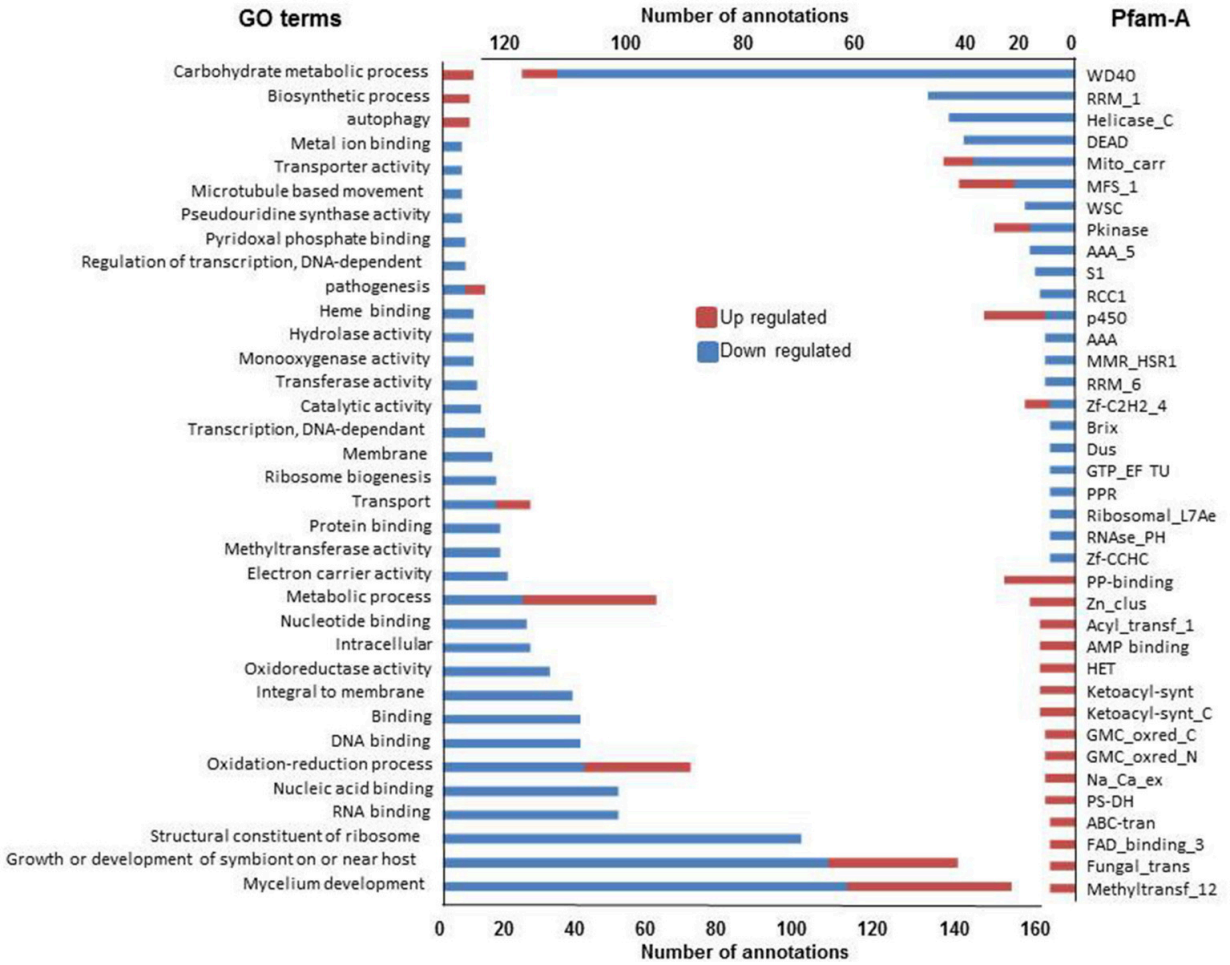
**GO terms and Pfam-A protein domains differentially expressed in all three non self conditions**. Only GO terms (left) and Pfam-A protein domains (right) found at least five times in one of the conditions in the up and down regulated gene sets are reported.

We first analyzed the 579 and 995 genes up or down regulated in all three conditions (Figure 5). As observed above, GO terms and Pfam-A annotations of genes up regulated in common are significantly less extensive than annotation of genes down regulated (Fisher test, *p* < 1^*e-*4^ in both cases). The most represented GO terms and Pfam-A domains associated with these gene sets, representing the common basis for response to non self, are presented Figure 5. Down regulated GO terms or Pfam-A annotations are essentially related to growth, development and protein synthesis. Up regulated GO terms or Pfam-A annotations are related to metabolic and catabolic process, secondary metabolism and autophagy (see below). The common basis of response to non self includes a growth arrest accompanied with reduction in protein synthesis, and induction of autophagy, a process associated with response to stress (Kroemer et al., 2010), and immune responses (Zhou et al., 2014; Shibutani et al., 2015).

**Figure 6:**
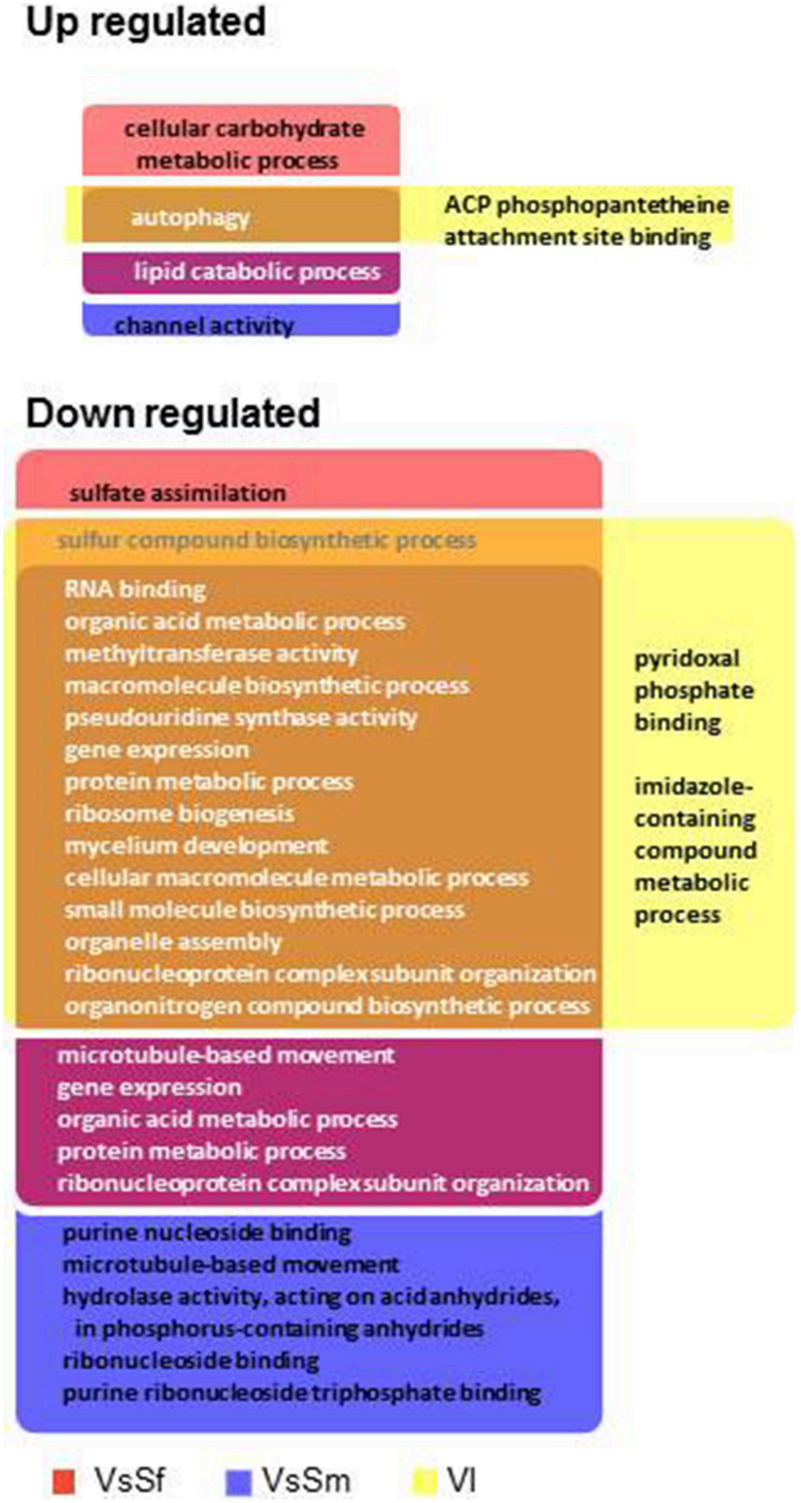
**Enriched GO terms in differentially regulated gene sets in presence of bacteria or during vegetative incompatibility for up or down regulated gene sets**.

We next identified and compared Biological Process and Molecular Function GO terms significantly enriched compared to their genomic representation (Fisher test, *p* < 0.001) in up or down regulated gene sets (Figure 6). We found few GO terms enriched with up regulated gene sets (from 8 to 18) and a single term, autophagy, is enriched in all three conditions (see below).Terms enriched in both VsSf and VsSm sets are concerned with lipid, carbohydrates and Reactive Oxygen Species (ROS) metabolic processes.

In contrast, we found many GO terms associated with down regulated gene sets (102–138), with 73 terms associated with all down regulated gene sets (Figure 6). They are essentially related to developmental processes such as ribosome assembly, RNA processing and protein synthesis and illustrate the transcriptional signature of the growth arrest observed in the three conditions. Note in addition that the 24 terms common to the VsSf and VsSm sets are essentially related to DNA metabolism.

Table 2 presents 25 Pfam-A domains significantly enriched or depleted in either of the conditions analyzed. The only Pfam-A domains enriched in all three up regulated gene sets correspond to proteins related to secondary metabolism generally involved in interspecific communication (see below). Domains enriched in presence of both bacteria include Heat Shock Proteins, transcription repressors of the NmrA family suggesting nitrogen repression (Andrianopoulos et al., 1998), and transmembrane ion transport. Note that all proteins displaying the Pfam-A domain PF01699 (Na-Ca-exchange) are predicted to be vacuolar which associated to the up-regulation of genes Pa_3_6420 and Pa_7_8320 encoding a regulator of V-ATPase and vacuolar ATP-synthase respectively, may indicate that regulating vacuolar pH is important in response to bacteria. Indeed *S. marcescens* is known to increase vacuolar pH when invading macrophage cells in culture (Fedrigo et al., 2011). Interestingly two domains related to transport of small solutes through membranes are specifically enriched in presence of *S. fonticola*. The enrichment of MFS transport often associated with drug extrusion could explain why *P. anserina* can survive to the presence of *S. fonticola*. Finally, the HET, LysM, and NACHT domains associated with incompatibility can be linked to NLR signaling related to defense functions (see below). Domains associated with down regulated genes in presence of bacteria are essentially associated with transport through nuclear membrane and RNA. Domains associated with incompatibility are also related to RNA binding and GTPase activity. Finally, note that WD40 repeat containing domains are highly represented in the three down regulated gene sets, while ankyrin repeat containing proteins are tightly regulated during VI but not in presence of bacteria. Four of the ankyrin containing proteins appear to be STAND proteins (see below), the remaining mostly correspond to proteins lacking a predicted function but usually these repeat domain proteins are involved in protein interactions with scaffolding functions (Javadi and Itzhaki, 2013).

**Table 2:** **Pfam-A protein domains enriched in at least one differentially expressed gene set**.

**Annotation**	**Name**	**Genome**	**VsSf**	**VsSm**	**VI**	**Brief description**
**UP REGULATED GENES**						
PF00153.22	Mito_carr	102	**40 (2,24)**	–	–	Membrane transport of small solute across mitochondrial and other membranes
PF07690.11	MFS_1	158	**51 (1,84)**	–	–	Membrane transport of small solutes including drugs
PF01699.19	Na_Ca_ex	16	**12 (4,28)**	**10 (4,16)**	–	Ion transport, Vacuolar homeostasis
PF08240.7	ADH_N	36	**18 (2,85)**	**19 (3,51)**	–	Alcohol deshydrogenase
PF00107.21	ADH_zinc_N	42	**19 (2,58)**	**20 (3,17)**	–	Alcohol deshydrogenase (partialy included in ADH-N)
PF00106.20	adh_short	79	28 (2,02)	**26 (2,19)**	–	Short chain deshydrogenase, reductase
PF07859.8	Abhydrolase_3	20	**12 (3,42)**	**12 (4)**	–	Hydrolases including lipases
PF00011.16	HSP20	6	**6 (5,7)**	**6 (6,66)**	–	HSP, Chaperonne in response to stress, pathogenesis in ustilago
PF01494.14	FAD_binding_3	38	**17 (2,55)**	**17 (2,98)**	–	Electron tranfer
PF00840.15	Glyco_hydro_7	7	5 (4,07)	**7 (6,66)**	–	Glycoside hydrolase
PF05368.8	NmrA	20	11 (3,14)	**10 (3,33)**	–	Negative transcriptional regulator, Nitrogen metabolite repression
PF00501.23	AMP-binding	49	**21 (2,44)**	**20 (2,72)**	–	AMP-binding in peptide synthases and four coumarate CoA ligase
PF13193.1	AMP-binding_C	18	10 (3,1)	**10 (3,7)**	–	All included in AMP-binding
PF00668.15	Condensation	30	**15 (2,85)**	**14 (3,11)**	–	Antibiotic synthesis in peptide synthase
PF00550.20	PP-binding	50	**25 (2,85)**	**21 (2,8)**	**24 (2,59)**	Prosthetic group, associated to PKS (includes four condensation)
PF00698.16	Acyl_transf_1	24	12 (2,85)	11 (3,05)	**15 (3,37)**	Acyl transferase, PKS
PF00109.21	ketoacyl-synt	24	12 (2,85)	10 (2,77)	**15 (3,37)**	PKS
PF02801.17	Ketoacyl-synt_C	23	11 (2,72)	9 (2,6)	**15 (3,52)**	PKS
PF14765.1	PS-DH	21	10 (2,71)	–	**15 (3,85)**	PKS
PF08659.5	KR	14	–	–	**11 (4,24)**	PKS
PF06985.6	HET	129	–	–	**62 (2,59)**	Vegetative incompatibility cell death
PF01476.15	LysM	31	–	–	**18 (3,13)**	Sugar binding, bacterial cell wall degradation
PF05729.7	NACHT	34	–	–	**17 (2,69)**	NLR oligomerization domain
PF00400.27	WD40	433	**13 (0,17)**	**17 (0,26)**	–	Repeat, protein ligand interaction
PF12796.2	Ank_2	167	**7 (0,24)**	**4 (0,16)**	**76 (2,46)**	Repeat, protein ligand interaction
**DOWN REGULATED GENES**						
PF13634.1	Nucleoporin_FG	12	**11 (4,85)**	**11 (5,28)**	–	Nuclear pore
PF00493.18	MCM	6	6 (5,29)	**6 (5,76)**	–	Replication licensing factors
PF14551.1	MCM_N	6	6 (5,29)	**6 (5,76)**	–	Comprised in MCM
PF00271.26	Helicase_C	77	**33 (2,27)**	**34 (2,54)**	–	Helicase, mostly associated to RNA
PF00270.24	DEAD	47	**28 (3,15)**	**29 (3,55)**	**22 (2,43)**	RNA helicase included in Helicase_C
PF00076.17	RRM_1	83	**39 (2,49)**	**35 (2,42)**	30 (1,88)	RNA binding
PF00153.22	Mito_carr	102	–	–	**53 (2,71)**	Membrane transport of small solute across mitochondrial and other membranes
PF01926.18	MMR_HSR1	12	–	–	**11 (4,77)**	GTPase
PF06985.6	HET	129	–	–	**5 (0,20)**	Vegetative incompatibility cell death
PF00400.27	WD40	433	**138 (1,69)**	**137 (1,82)**	**122 (1,47)**	Repeat, protein ligand interaction
PF12796.2	Ank_2	167	**10 (0,32)**	**9 (0,31)**	**3 (0,09)**	Repeat, protein ligand interaction

*Along with the Pfam-A domain accession numbers and names are indicated the number of annotations in the genome and in the differentially expressed gene sets, the numbers in brackets indicating the enrichment factor (Two tails Fisher's test, p < 0.001 in bold, otherwise p < 0.01). A brief description of the domain function is also presented*.

### Secreted proteins

Secreted proteins are involved in interspecies communication in situation of host pathogen or host symbiont interactions. We analyzed the expression of the 801 genes encoding predicted secreted proteins from *P. anserina*, and found no bias for genes encoding CAZyme, proteases or lipases, three of the main classifications for secreted proteins (Pellegrin et al., 2015). However, genes encoding Small Secreted Proteins (SSP, protein of less than 250 aa) are overrepresented in the up regulated gene set in response to bacteria, but not during the VI reaction for genes with a fold change over 4 (Table 3). SSPs in the context of host pathogen or host symbiont interactions act on the host cell to modify its behavior, and are thus considered fungal effectors. It appears that they can also be involved in the fungal reaction to heterospecific non self, possibly acting directly on the bacterial cells. In that respect it is relevant to note that within the expressed SSP encoding genes are *idi1, idi2*, and *idi3* previously identified in the context of VI. IDI2 protein is homologous to the victoriocin displaying an antifungal activity in the species *Chochliobolus victoriae* (de Sá et al., 2010).

**Table 3:** **Expression of Small Secreted Protein encoding genes**.

	**VsSf**	**VsSm**	**VI**
FC > 2	64/1.2/ns	56/1.2/ns	57/1/ns
FC > 4	52/1.8/5^*e-*5^	46/1.8/9^*e-*5^	45/1.3/ns
FC > 6	38/2.5/8^*e-*10^	30/2.5/2^*e-*7^	25/1.3/ns
FC > 8	20/2.5/2^*e-*6^	17/2.8/2^*e-*6^	11/1/ns

*For each reaction the number of SSP encoding genes, the enrichment compared the expected number, and the Fisher's two tail test p-value are indicated (Number/Enrichment/p-value), depending of the fold change (FC) threshold level. Ns, non-significant*.

### Autophagy contributes to survival against bacterial non self

**Figure 7:**
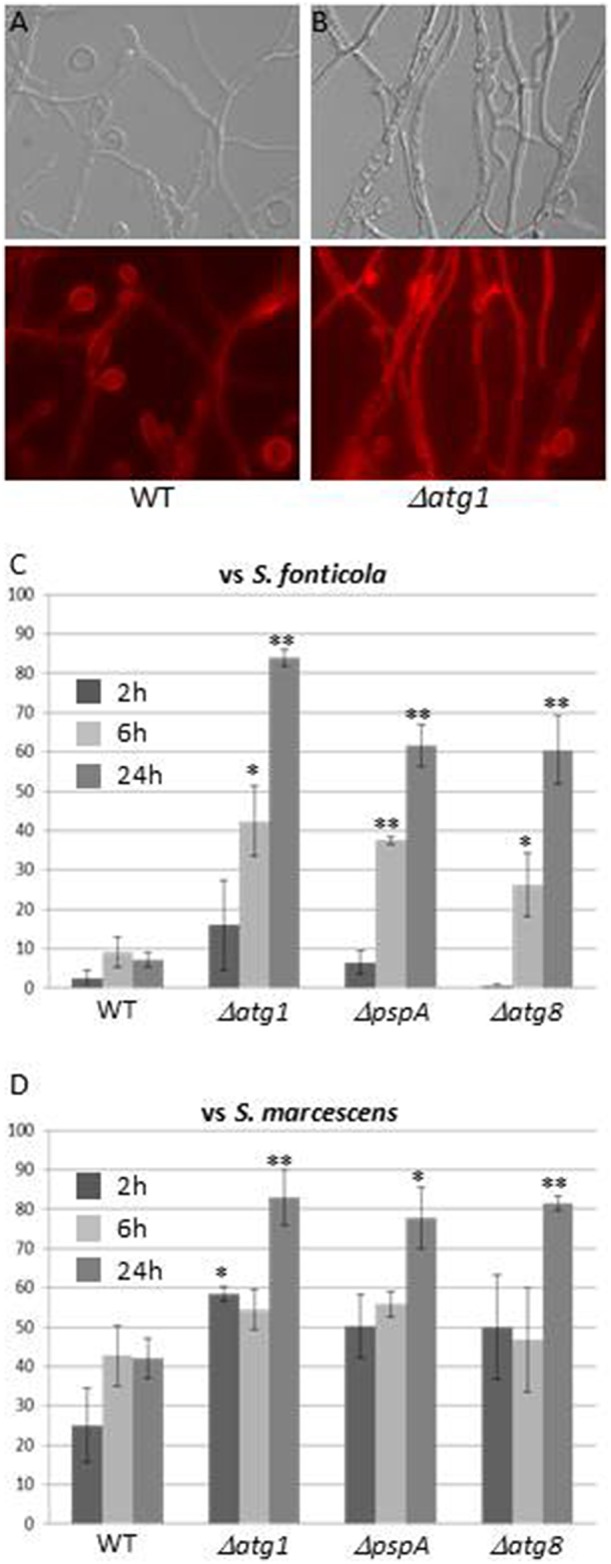
**Autophagy exerts a pro-survival function in response to bacteria. (A,B)** Light or *atg1* autophagy mutant ∆ Evans blue staining of WT **(A)** or **(B)** 6 h after transfer onto a *S. fonticola* seeded plate. **(C,D)** Cell death level measure *atg8* autophagy mutants after transfer onto *S. ∆pspA*, and ∆*atg1*, ∆ for WT, fonticola **(C)** or *S. marcescens* **(D)** seeded plates. Cell death was estimated from 10 different pictures of three independent experiments for each time point (two tailed *t*-test, ^*^p < 0.05, ^**^p < 0.001).

Autophagy, the only GO term associated with all three responses, is generally associated to stress responses (Kroemer et al., 2010), and has also been described as essential to immune response in plants and animals (Zhou et al., 2014; Benoit et al., 2015; Shibutani et al., 2015). Genes encoding components of the *S. cerevisiae* autophagy machinery were recently reviewed (Feng et al., 2014). Eighteen have orthologs in *P. anserina*, 12, 10, and 12 of which are up regulated in the VsSf, VsSm, or VI gene sets while none is down regulated (Additional file 4). The role of autophagy as a cell death or survival mechanism seems to depend on the context and model (Dickman and Fluhr, 2013). Autophagy has been extensively studied in *P. anserina*, in particular in the context of VI where it exerts a pro-survival function, and was hypothesized to restrict spread of death signals from the heterokaryotic cell (Pinan-Lucarré et al., 2005, 2007). We transferred three *P. anserina* autophagy mutants, Δ*Pa-ATG1*, Δ*Pa-ATG8* (Pinan-Lucarré et al., 2005), and Δ*pspA* (Paoletti et al., 2001) involved at different stages of the process onto *S. fonticola* or *S. marcescens* seeded plates. In presence of both bacteria, autophagy mutant cell death rapidly reaches high levels not attained by WT strain (60–80%) (Figure 7). This is particularly clear in transfer to *S. fonticola* seeded plates, while in response to *S. marcescens* level of WT cell death is initially slightly inferior to that of the autophagy mutant strains. These results indicate that as for VI, autophagy exerts a pro-survival function in response to bacteria. This pro-survival function of autophagy is the clearest in response to *S. fonticola*, but seems attenuated in response to *S. marcescens*. Interestingly *S. marcescens* is known to be able to alter the pH of autophagic like vesicles in mammalian cells (Fedrigo et al., 2011) and one could speculate that such action renders autophagy less efficient in *P. anserina* as well.

### Reactive oxygen species

**Figure 8:**
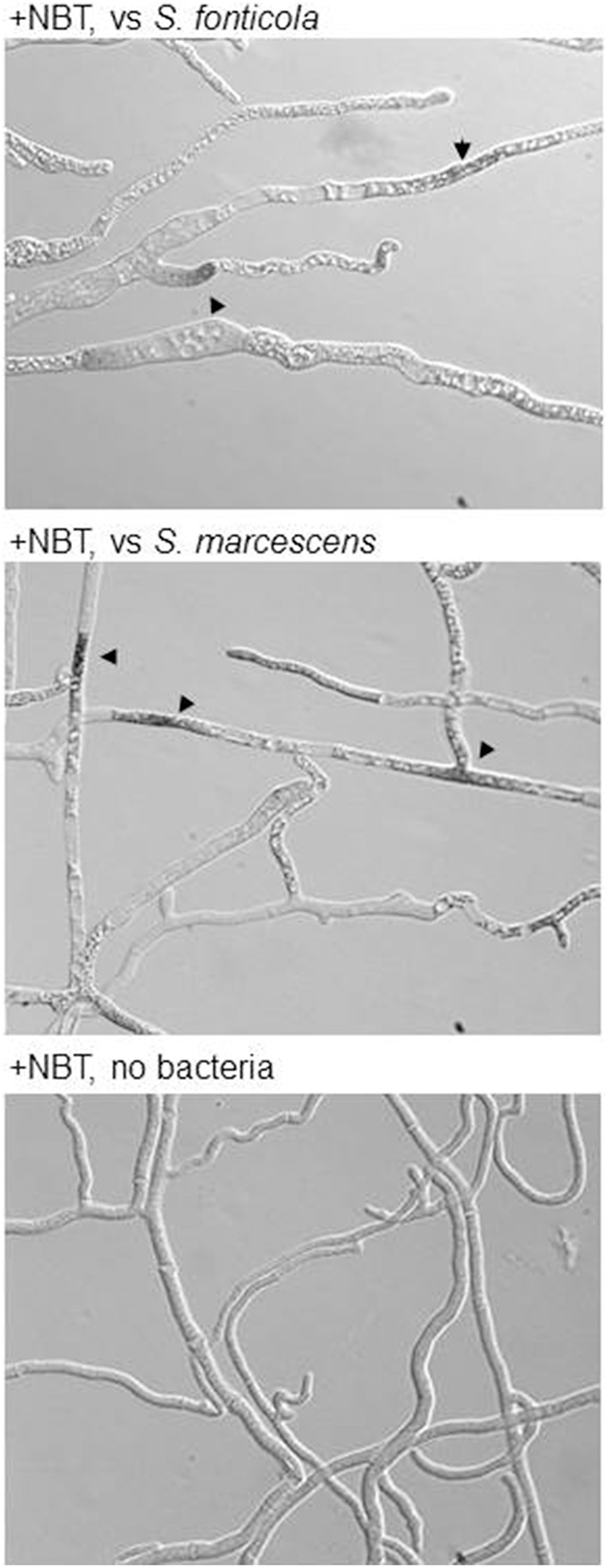
***P. anserina* is submitted to an oxidative stress in response to bacteria:** Upon transfer for 2 or 6 h onto *S. fonticola* or *S. marcescens* seeded plates ROS are detected in *P. anserina* dead or dying cells (arrowheads) that are not detected after transfer to bacteria free plates.

ROS are often produced in the context of host/pathogen interactions either as a defense mechanism by the host or as a debilitating factor by the pathogen (Gessler et al., 2007). We have compiled *P. anserina* genes encoding ROS producing enzymes or acting as antioxidant agents (Additional file 5). It is clear that in reaction to bacteria, expression of antioxidant components is stimulated, which is not the case during the VI reaction, while expression of the main ROS producing enzymes (PaNOX1-3) is not stimulated. Using a probe reactive to ROS we indeed observed their accumulation in dead or dying cells in presence of bacteria (Figure 8). These observations suggest that *P. anserina* is confronted to an oxidative stress generated by the presence of the bacteria. It was already observed that *P. anserina* accumulated peroxides in response to certain fungal species but not during VI (Silar, 2005).

### Secondary metabolite

Secondary metabolites constitute central elements of the chemical arsenal for inter organismal communications, and fungal secondary metabolite production has been shown to be stimulated by bacteria in fungi (Brakhage, 2013). Thirty five secondary metabolite gene clusters comprising 470 genes have been identified in *P. anserina* (Bills et al., 2013). In agreement with the Pfam-A protein domain analysis, they are over represented in the three up regulated gene sets (Fisher's test, *p* < 0.005, Additional file 6), with a total of 126, 122, and 142 genes in the VsSf, VsSm and VI up regulated gene sets. We also observed that 33, 31, and 34 clusters having at least one gene up regulated, and 22, 22, and 21 have at least three genes up regulated in the VsSf, VsSm, or VI gene sets (Additional file 6). Only 52 genes (including 8 out of 17 expressed polyketide synthase encoding genes) are up regulated in all three conditions suggesting that the final secondary metabolites produced in VI or in response to bacteria likely differ.

### Signaling

In *P. anserina* we identified 77 such NLR encoding genes (Dyrka et al., 2014). They are not transcriptionally regulated in presence of bacteria, but they are overrepresented (Fisher test, *p* < 0.001) in the up regulated genes during the VI reaction (Table 4) as already reported (Bidard et al., 2013). In *P. anserina* NLR proteins, 7 N-terminal domains with a known annotation were identified, three of which (HET, HeLo, HeLo-like) known or suspected to induce cell death in *P. anserina* (Paoletti and Clavé, 2007; Daskalov et al., 2012, 2015). Two N terminal domains are related to lipase, one (sesB) altering growth in *Nectria haematococca* (Graziani et al., 2004), the other (Patatin) controlling PCD and defense in plants (Kumar and Kirti, 2012; Kim et al., 2014). All these domains are also found in non-NLR proteins (Daskalov et al., 2012). As these effector domains are not all Pfam-A annotated, we used an in-house annotation pipeline to identify them in all *P. anserina* cds, and examined expression of the corresponding genes (Table 4). For each domain they are expressed in higher numbers during VI than in response to bacteria. Taken altogether they appear over-represented in VI gene set (*p* < 0.001). This remains true when the two most represented HET and HeLo like domains are subtracted from the analysis (*p* < 0.05). Overall VI conditions result in expression of NLR N-terminal or stand-alone effector domains known to induce cell death in *P. anserina*. Note that HeLo and HeLo-like domains act as pore forming toxins through insertion in biological membranes (Mathur et al., 2012; Seuring et al., 2012), and could thus also be active on membranes of pathogens in addition to *P. anserina*. Indeed HeLo domain is known to induce cell death when expressed in yeast cells (Taneja et al., 2007). The same could be true for the other N terminal effector domains.

**Table 4:** **NLR and STAND alone effecter domains gene expression**.

**Domain**	**Function**	**Cell death**	**NLR**				**Non NLR**			
			**Genome**	**Up VsSf**	**Up VsSm**	**Up VI**	**Genome**	**Up VsSf**	**Up VsSm**	**Up VI**
GOOD-BYE	–	–	10	2 (2)	1 (1)	6^*^	4	1	0	2
HeLo-like	Pore forming	Suspected	4	1 (1)	0	3^*^	20	4 (2)	4 (3)	18^**^
HeLo	Pore forming	Yes	–	–	–	–	7	3 (3)	3 (3)	4
HET	–	Yes	5	0	0	2	124	16 (9)	19 (13)	61^**^
Patatin	Lipase	Suspected	1	0	0	2	9	5* (2)	5* (1)	3
PNP_UDP	Sugar metabolism	–	1	0	0	2	5	0	0	3
REL-SPO	ppGpp metabolism	–	1	0	0	1	–	–	–	–
SesB	Lipase	Suspected	12	0	0	3	24	4 (2)	4	7
Unk	–		39	2	1	10	–	–	–	–
total			73	5	2	29	193	33	35	98

The numbers of NLR and standalone effecter domain encoding genes up regulated in response to non self are reported. For responses to bacteria numbers of genes also regulated during VI are indicated in brackets. Fisher tests were conducted to identify genes significantly enriched in comparison to their representation in the genome (

* *p < 0.05*,

** *p < 0.001)*.

Histidine kinase (HK) function as sensors for external or internal stimuli and are able to activate response pathways either directly, or in two component system via phosphotransfer protein (HPT) (Schaller et al., 2011). HK encoding genes are over represented in up regulated gene sets as described in details in Additional file 7.

### Transcription factors and promoters

*P. anserina* genome encodes for 216 putative transcription factors. TF encoding genes are under-represented in the down regulated gene sets for all three conditions (2.1 folds, *p* = 1.5^*e-*3^ for VsSf, 2.4 folds *p* = 7.2^*e-*4^ for VsSm, 2.7 folds *p* = 9.1^*e-*5^ for VI). Of the 30 down regulated TF genes, 12 have orthologs with known functions that are essentially related to development. Of the 84 TF up regulated, only 13 have orthologs with known functions, indicating that TF associated with response to non self remain largely uncharacterized (Additional file 8). This figure also presents the overlap between up regulated TF genes between all three conditions. Within the TF genes up regulated in all three conditions is *Atf1*, a general stress responsive transcription factor (Lawrence et al., 2007). One of the two TF over-expressed in both VsSm and VI conditions, the two conditions resulting in *P. anserina* cell death, is *idi-4* whose overexpression initiates a cell death reaction comparable to the VI reaction (Dementhon and Saupe, 2005). However, deletion of *idi4* does not prevent cell death during VI or in presence of bacteria (not shown). A single TF expressed in presence of both bacteria has an ortholog with a known function in secondary metabolism control. Interestingly, TF genes are significantly over-represented only in the VsSf up regulated gene set (*p* = 2^*e-*4^), the only condition where *P. anserina* survives, suggesting that the ability to stimulate the expression of a large number of pathways may contribute to the resistance to bacteria. They include genes with orthologs encoding TF involved in response to stresses, regulating expression of secondary metabolite synthesis, and orthologs of *N. crassa* WC-1 and WC-2 (WC-1 and WC-2 are also up regulated in presence of *S. marcescens* but below the FC > 2 threshold) involved in response to blue light and regulation of the circadian clock (Chen et al., 2010, see below).

We analyzed 500 bp promoter sequences using Scope (Carlson et al., 2007) to identify putative cis regulating sequences and found some that are significantly enriched or depleted in the up or down regulated gene sets (Additional file 8). Interestingly, the binding site ATGAnTCAT identified as the IDI4 target during VI (Dementhon and Saupe, 2005) is specifically enriched in the genes up regulated in presence of bacteria. We found a single GO term (autophagy) associated with putative cis regulating sequence kACGTCAb in the up regulated gene set, while all sequences enriched in down regulated gene sets are associated with GO terms, essentially related to translation and ribosomal proteins.

### Comparison to other responses

Fungal transcriptional response in BFI conditions have been investigated in a limited number of cases. Numbers of fungal genes up or down regulated in response to bacteria vary greatly depending on the nature of the interaction and the experimental set ups. For instance, few genes are regulated in *A. niger* confronted to *C. fungivorans* (Mela et al., 2011) or in *L. bicolor* confronted to *P. fluorescens* (Deveau et al., 2015), while high numbers or regulated genes are reached in *A. niger* confronted to *Bacillus* (Benoit et al., 2015) or in *R. solani* confronted to *Serratia proteamaculans* or *S. plymuthica* (Gkarmiri et al., 2015).

Recently was reported the transcriptional response of the basidiomycete plant pathogen *R. solani* to bacterial species *S. proteamaculans* and *S. plymuthica* used as bio-control agents (Gkarmiri et al., 2015). In both of these BFIs fungal growth toward the bacteria stops and the fungal reaction including cell swelling and increased septation appears similar to what we observed in *P. anserina* in response to *S. marcescens* and *S. fonticola*. We thus compared the fungal transcriptional responses. From the 3228 *R. solani* genes found to be up or down regulated in response to *Serratia* species, we found 712 genes with an ortholog in *P. anserina* as identified by a reciprocal best hit approach. We then counted the orthologous gene pairs up or down regulated in response to bacteria in both fungal species (Table 5). Orthologous gene pairs up regulated by *Serratia* species in both fungi are over represented in the set of up regulated *R. solani* genes. As well, orthologous gene pairs down regulated in response to bacteria in both fungi are over represented in the set of *R. solani* genes down regulated in response to bacteria. Thus, *R. solani* and *P. anserina* up and down transcriptional responses to *Serratia* species overlap. However, the overlap is significantly more important for the down regulation than for the up regulation as on average the proportion of down regulated orthologous gene pairs represents twice that of up regulated genes. For example 60 out of 220 *R. solani* up regulated genes in response to *S. plymuthica* have orthologs in *P. anserina*, while 114 out of 224 *R. solani* genes down regulated in response to the same bacteria have orthologs in *P. anserina*. Pfam-A protein domain annotation and GO term associated with the orthologous gene pairs down regulated in both fungal species are related to growth, development, gene expression and protein synthesis, while up regulated genes are associated with metabolic processes (Additional file 8). Note that within the orthologous gene pairs regulated in both fungal species we found the expected number of genes also regulated during VI (Table 5). Overall, these results are in accordance with the idea that fungal responses to antagonistic bacteria include a growth arrest associated with the down regulation of genes required for development, DNA, RNA, and protein synthesis. It also includes the stimulation of expression of a common set of genes completed with lineage specific genes.

**Table 5:** **Responses of *P. anserina* and *R. solani* to *Serratia* species overlap: For each *R. solani* up or down regulated genes sets in response to *S. Plymuthica* or *S. proteamaculans* with orthologs in *P. anserina* (numbers in brackets) we counted genes up or down regulated in response to bacteria in *P. anserina* or during VI, calculated the enrichment compared to the expected number, and the Fisher two tail *p*-value**.

***R. solani*** **vs**. ***S. plymuthica*** **AS13**			***R. solani*** **vs**. ***S. proteamaculans*** **S4**		
*R. solani* up vs. AS13 (220)	*R. solani* down vs. AS13 (224)	Down/Up	*R. solani* up vs. S4 (279)	*R. solani* down vs. S4 (352)	Down/Up
Up VsSf: 60/1.5/0.02 /*10*	Down VsSf: 114/2.9/7^*e-*14^/*79*	1.9/3.8^*e-*7^	Up VsSf: 78/1.6/0.006/*19*	Down VsSf: 178/2.9/1.6^*e-*20^ *125*	1.8/1.6^*e-*8^
Up VsSm: 55/1.6/0.02 /*12*	Down VsSm: 107/3/2.3^*e-*13^ *79*	1.9/7.3^*e-*7^	Up VsSm: 68/1.6/0.01/*19*	Down VsSm: 171/3/8.8^*e-*21^ *124*	2/1.1^*e-*8^
Up VI: 20/0.5/0.004	Down VI: 87/2.1/1.1^*e-*6^	4.2/4.8^*e-*9^	Up VI: 36/0.7/ns	Down VI: 144/2.2/4.5^*e-*11^	3.2/7.4^*e-*9^

*(Number/Enrichment/p-value). We also report for orthologous gene pairs regulated in both fungal species the number of genes regulated during VI (Numbers in italics). The Down/Up columns reports the ratio of the down to up enrichment factors, and the Fisher two tail test p-value*.

In a *P. anserina* colony three distinctive regions of the mycelium can be distinguished corresponding to an outward region were growth occurs, an intermediate region were most of the sexual development takes place, and a central region were growth has stopped. Gene expression in these three zones has been examined and compared to each other (Bidard et al., 2012). We compared differential gene expression in *P. anserina* in response to bacteria or during incompatibility to gene expression in the different regions of the mycelium obtained by Bidard and colleagues. Genes expressed at a higher level in the growing part of the mycelium (zone 1) are enriched in genes down regulated in presence of bacteria or VI, which was expected as these are essentially related to development and growth. Perhaps more surprisingly, genes expressed to a higher level at the center of the fungal colony (in stationary phase) are significantly enriched in genes up regulated in presence of bacteria or during VI, and depleted in genes down regulated in the same conditions (Additional file 10A). These data suggest that when *P. anserina* enters stationary phase, it expresses genes required in reaction to non self, possibly preserving older mycelium from being exploited by invasive microorganisms.

*P. anserina* orthologs of *N. crassa WC-1* and *WC-2* are up regulated in response to bacteria. In *N. crassa* transcription factors WC-1 and WC-2 form the White Collar Complex (WCC) required for gene expression in response to blue light and to regulate circadian rhythm (Chen et al., 2010). We observed a significant overlap between response to bacteria in *P. anserina* and WCC dependent response to blue light in *N. crassa* as reported in Additional file 10B. We also compared *P. anserina* transcriptional responses to VI in *N. crassa* and found a significant but limited overlap between VI conditions as already reported (Additional file 10C, Bidard et al., 2013).

### A multilayered response to non self

In plants and animals, induction of the full innate immune response results from the detection of multiple signals, including detection of conserved pathogen associated molecular patterns (PAMP) through transmembrane receptors, and danger signals detected by cytoplasmic NLRs (Stuart et al., 2013). In our experimental set up, responses to bacteria initiated without any contact thus detection likely involves diffusible molecules. For instance bacteria are known to produce outer membrane vesicles composed essentially of lipopolysaccharides, a classical PAMP to which *P. anserina* reacts (unpublished). Detection could also occur through danger signals generated by diffusible toxins. In contrast, VI condition is initiated by cytoplasmic NLR thought to mimic detection of a cytoplasmic danger signals (Paoletti and Saupe, 2009; Bastiaans et al., 2014).

**Figure 9:**
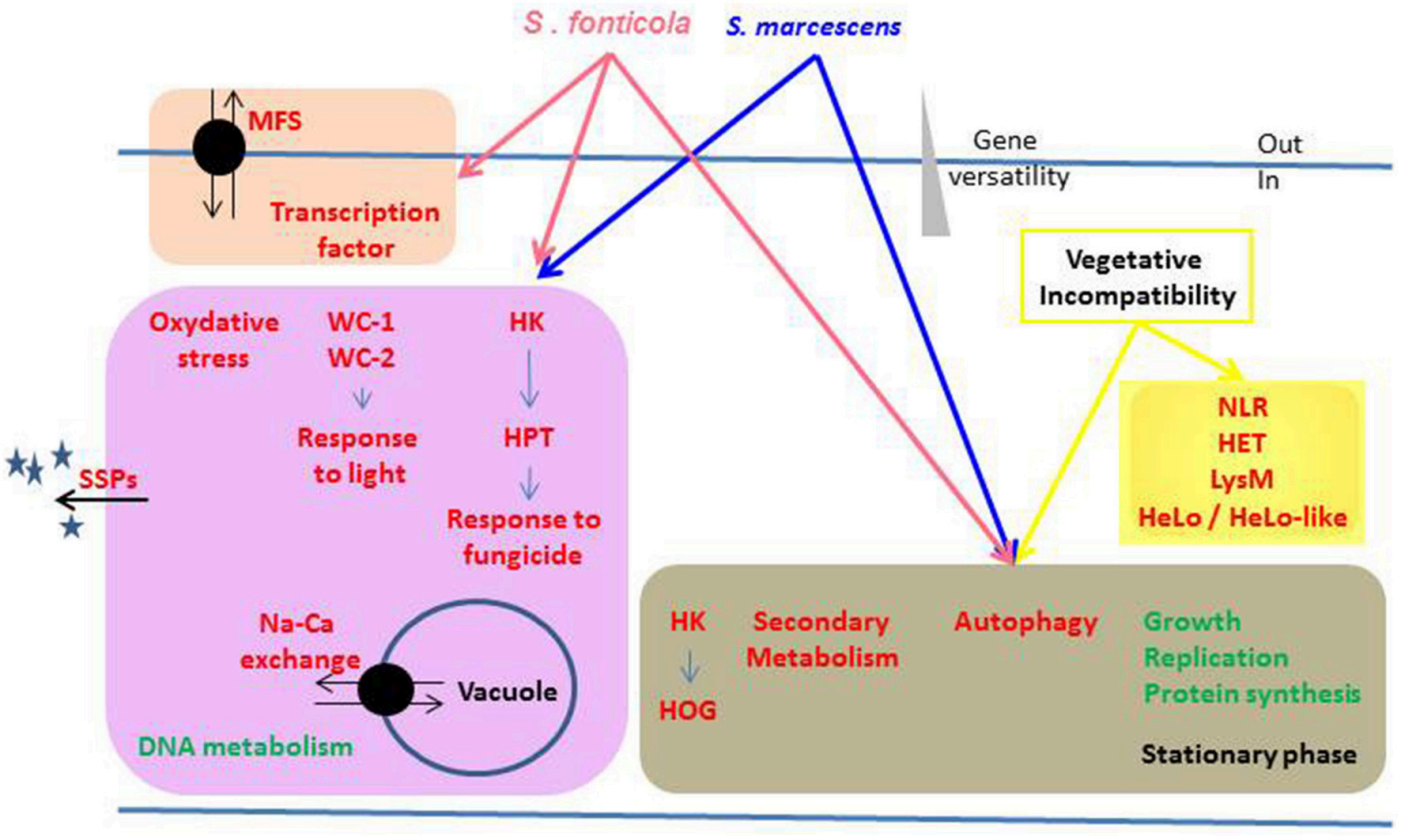
**Overview of the biological functions regulated in response to non self**. Biological functions or pathways significantly enriched in up (red) or down (green) regulated gene sets in response to different non self signals are represented. Functions differentially regulated by the same non self signals are grouped in colored boxes, arrows indicating the inducing conditions.

From Pfam-A or GO annotations we identified biological functions up or down regulated in response to non self that are summed up in Figure 9. All three conditions result in a growth arrest that is reflected by functions encoded by the down regulated genes mostly related to protein synthesis and growth. The conserved up regulated functions common to all three conditions are common in responses to stress (HK signaling, autophagy) and biotic interactions (autophagy and secondary metabolites). Interestingly, all functions specifically up regulated in response to one or both bacteria (SSPs, response to oxidative stress and light response, and response to fungicide through HK signaling, MFS transporters, vacuolar pH regulation) could promote fungal cell survival. For instance, *P. anserina* expresses more transcription factors and MFS transporters and survives better in response to *S. fonticola* than in response to *S. marcescens*, and difference in gene expression and survival may be dependent on the arsenal of bacterial pathogenicity effecters they produce (Guichard et al., 2014). In contrast, most functions specifically up regulated in the VI reaction seem to promote fungal cell death. The HET domain is an activator of a VI-like cell death (Paoletti and Clavé, 2007), HeLo and HeLo like domains act as pore forming toxins (Daskalov et al., 2015). Except for a few NLR controlling VI (Saupe et al., 1995; Espagne et al., 2002; Choi et al., 2011) fungal NLR functions have not been characterized but they often display HET or HeLo N-terminal effector domains. LysM domain function has not been defined in fungi, but it is known to bind sugars and promote immune responses in plants (Miya et al., 2007; Gimenez-Ibanez et al., 2009; Tanaka et al., 2013; Akcapinar et al., 2015). From these observations, one might propose that like plants and animals, fungi are endowed with a multilayered innate immune system that can be sequentially activated depending on the signals detected. Detection of conserved bacterial PAMPs from long range may initiate a robust and fairly conserved response whose aim is to promote fungal survival while making life of the pathogen difficult. Indeed most fungi will encounter all sorts of bacteria during their life time which will require a global response to keep them at bay. This would be comparable to the plant PTI (Jones and Dangl, 2006). Then, adapted bacteria may be able to subdue this first line of defense through the action of specifically evolved effectors. Detection of the resulting danger signals would then initiate the ultimate fungal response by triggering a localized cell death reaction through NLR signaling to restrict further invasion of the entire mycelium in a response similar to the plant Hypersensitive Response (Jones and Dangl, 2006). Positive feedback on NLR encoding genes, and standalone N-terminal effecter genes, could ensure robustness of the response by initiating multiple death pathways thereby preventing pathogens from developing inhibitors of a single pathway.

As for plants and animals responses to pathogenic non self, simply considering the number of genes commonly up or down regulated in response to bacteria or during VI reveal that the different layers of the immune response largely overlap in *P. anserina*. In addition, in all three conditions, versatile genes thought to define adaptive traits (Wapinski et al., 2007) are over-represented in up regulated gene sets. This trend is particularly clear in the VI conditions that result in the activation of additional genes in the most versatile categories. A response to non self thus seems to induce mostly adaptive genes, however the detection of conserved PAMPs initiates a more general response than detection of intracellular signals through lineage specific NLR receptors to detect adapted pathogens and require a more specific response.

## Author contributions

ML, WD, AB, and MP designed and conducted the experiments, SS and MP contributed to the writing of the paper.

## Funding

This work was funded by ANR grant “MYKIMUN” number ANR 11 BSV3 109 01.

### Conflict of interest statement

The authors declare that the research was conducted in the absence of any commercial or financial relationships that could be construed as a potential conflict of interest.
