# Supplementary material for: Overlapping Podospora anserina Transcriptional Responses to Bacterial and Fungal Non Self Indicate a Multilayered Innate Immune Response
Source: Front Microbiol. 2016 Apr 19;7:471. doi: 10.3389/fmicb.2016.00471 (PMC4835503; doi:10.3389/fmicb.2016.00471)
Supplement: Supplementary file 2 [file Table2.DOC]

| Complex | S. cerevisiae | P. anserina | VsSf | VsSm | VI |
| --- | --- | --- | --- | --- | --- |
|  | Atg1 | Pa_7_10890 | up | up | up |
| ATG1 | Atg13 | Pa_0_1500 | up | up | Up |
| complex | Atg17 | Pa_7_4900 | _ | _ | _ |
|  | Atg29 | Pa_1_19480 | _ | _ | _ |
|  | Atg31 | _ | _ | _ | _ |
| Atg9 | Atg2 | Pa_2_12950 | up | up | up |
| cycling | Atg9 | Pa_5_5550 | up | up | up |
| system | Atg18 | Pa_6_3970 | _ | _ | up |
|  | Vps34 | Pa_1_20030 | _ | _ | _ |
| PtdIns3K | Vps15 | Pa_6_1830 | up | _ | up |
| complex | Vps30/atg6 | Pa_1_11020 | up | up | up |
|  | Atg14 | _ | _ | _ | _ |
|  | Atg8 | Pa_3_5250 | up | up | up |
| Atg8 Ubl | Atg7 | Pa_2_1770 | up | up | up |
| Conjugation | Atg3 | Pa_1_20610 | up | up | up |
| system | Atg4 | Pa_5_5430 | up | up | up |
|  | Atg12 | Pa_4_7460 | up | _ | _ |
| Atg12 ubl | Atg7 | Pa_2_1770 | Up | up | up |
| conjugation | Atg10 | _ | _ | _ | _ |
| system | Atg16 | Pa_7_6440 | _ | _ | _ |
|  | Atg5 | Pa_2_160 | _ | _ | _ |

Adapted from Feng Y., He D., Yao Z. and Klionsky D.J.in Cell Research (2014) 24:24-41
