# Supplementary material for: Overlapping Podospora anserina Transcriptional Responses to Bacterial and Fungal Non Self Indicate a Multilayered Innate Immune Response
Source: Front Microbiol. 2016 Apr 19;7:471. doi: 10.3389/fmicb.2016.00471 (PMC4835503; doi:10.3389/fmicb.2016.00471)
Supplement: Supplementary file 5 [file Table5.DOC]

**Supplementary figure 7: Histidine Kinase Signalling**

Histidine kinase (HK) function as sensors for external or internal stimuli and are able to activate response pathways either directly, or in two component system via phosphotransfer protein (HPT) . In filamentous fungi HK often respond to alteration of cell wall, osmotic or oxidative stress and can activate the High Osmolarity Glycerol (HOG) MAP kinase. *P. anserina* genome encodes for 12 HK, six of which are up regulated in presence of bacteria along with the single HPT encoding gene (Additional file 6). In VI 5 of these HK genes are up regulated. In *A. nidulans* the HK NikA is the target of the fungicide (fludioxonil) that activates transcription of 283 genes , 248 of which have orthologues in *P. anserina*. These orthologues are overrepresented (2.1 folds, Additional file 6) in up regulated VsSf and VsSm gene sets but not during VI. We also note that the gene Pa_1_23930 encoding a MAP kinase orthologous to *HogA*, and Pa_2_1280 encoding an orthologue of the transcription factor ATF1 controlled by the HOG pathway are up regulated in response to bacteria and during VI. Although HK are known to cross talk with many pathways, it is tempting to hypothesize that in response to bacteria HK Pa_3_8500 and Pa_1_10100 activate the HPT Pa_3_7200 and a response similar to the response to fludioxonil in a HOG independent pathway, while other HK signals stress response through the HOG pathway.
