# Supplementary material for: Overlapping Podospora anserina Transcriptional Responses to Bacterial and Fungal Non Self Indicate a Multilayered Innate Immune Response
Source: Front Microbiol. 2016 Apr 19;7:471. doi: 10.3389/fmicb.2016.00471 (PMC4835503; doi:10.3389/fmicb.2016.00471)
Supplement: Supplementary file 8 [file Table8.DOC]

**Supplementary figure 10A: Comparison of the transcriptional response to non self to the *P. anserina* transcriptome in stationary phase**

In their study, Bidart and co-authors (Fungal Genetics and Biology 49, p643-652, 2012) divided a *P. anserina* fungal colony in three phenotypically distinct regions. Zone 1 at the edge of colony corresponds to growing hyphae, zone 2 corresponds to the portion of mycelium where most of the sexual development takes place and Zone 3 corresponds to a portion of the mycelium in stationary phase. They identified 7 clusters of differentially expressed genes across the fungal colony.

We found that genes differentially expressed in response to non self are over-represented in genes highly expressed in zone 3 at the center of the colony while genes down regulated in response to non self are underrepresented in the same central region. Inversely, genes repressed in response to non-self are over-represented in genes highly expressed in zone one.

|  |  | Up VsSf | | Up VsSm | | Up VI | | Down VsSf | | Down VsSm | | Down VI | |
| --- | --- | --- | --- | --- | --- | --- | --- | --- | --- | --- | --- | --- | --- |
| 7 clusters | Zone of maximal  expression | Nb | E (p) | Nb | E (p) | Nb | E (p) | Nb | E (p) | Nb | E (p) | Nb | E (p) |
| Down 1 (356) | 1 | 56 | _ | 55 | _ | 40 | _ | 134 | 2.2 (1.9^e-9^) | 123 | 2.3 (1.9^e-9^) | 134 | 2.1 (1^e-8^) |
| Down 2 (84) | 1 | 29 | 1.9 (0.02) | 27 | 1.9 (0.03) | 14 | _ | 10 | _ | 8 | _ | 18 | _ |
| Up-down 1 (29) | 2 | 0 | _ | 0 | _ | 0 | _ | 5 | _ | 4 | _ | 4 | _ |
| Up-down 2 (107) | 2-3 | 28 | _ | 23 | _ | 55 | 2.7 (8^e-7^) | 18 | _ | 9 | _ | 5 | 0.25 (2.3^e-3^) |
| Up 1 (33) | 3 | 10 | _ | 8 | _ | 13 | _ | 10 | _ | 7 | _ | 13 | _ |
| Up 2 (622) | 3 | 335 | 3.1 (1.4^e-42^) | 298 | 2.8 (1.5^e-31^) | 221 | 2 (5^e-12^) | 35 | 0.3 (2.5^e-11)^ | 20 | 0.2 (5.9^e-11^) | 30 | 0.3 (1.2^e-13^) |
| Up 3 (187) | 3 | 59 | 1.8 (2.6^e-3^) | 54 | 1.7 (6.^4e-3^) | 34 | 1.9 (6.1^e-4^) | 35 | _ | 20 | _ | 30 | _ |


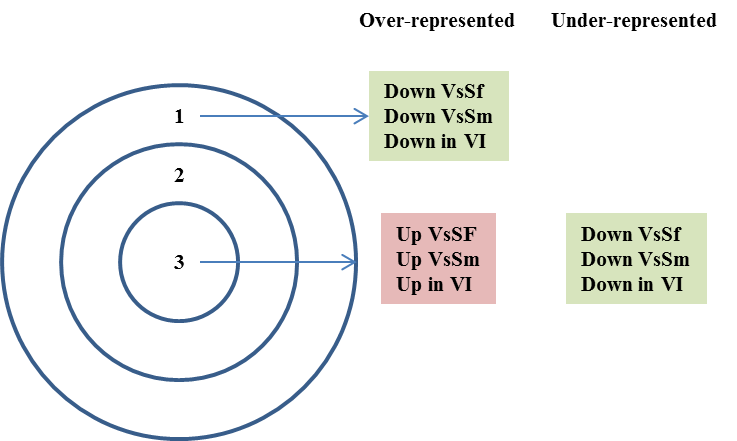


**Supplementary figure 10B :** Comparison of the transcriptional response to blue light in *N. crassa* and transcriptional response to non self in *P. anserina*.

*P. anserina* orthologues of *N. crassa WC-1* and *WC-2* are up regulated in response to bacteria. In *N. crassa* transcription factors WC-1 and WC-2 form the White Collar Complex (WCC) required for gene expression in response to blue light and to regulate circadian rhythm . The 1000 genes most up or down regulated by exposure to blue light have been identified . We analyzed the expression of their *P. anserina* orthologues in response to non self and found a clear overlap between the transcriptional response to bacteria in *P. anserina* and response to blue light in *N. crassa* for the up regulated gene sets, while no significant overlap was found with genes up regulated by VI (additional file 9B). In another study light responsive genes in *N. crassa* were divided in early and late responsive genes . Genes up regulated in presence of bacteria correspond essentially to late light responsive genes (additional file 9B). Functions associated with the corresponding genes include ROS detoxification, secondary metabolite production, glycoside hydrolases or transcription factors. Genes down regulated by blue light in *N. crassa* and genes down regulated by all three non self responses also clearly overlap (additional file 9B). Note that a relation between circadian rhythm and immune function has been proposed in plants and animals . Circadian regulation of genes involved in defense against pathogens could prepare *P. anserina* to bacteria whose activity is stimulated by exposure to light, including pathogenic bacteria . A link between response to light and response to ROS has also been proposed in *N. crassa* .

In their study, Wu and co-authors (G3, Vol 4, p1731-45, 2014) identified the 999 genes most up or down regulated after exposing *N. crassa* to blue light which have respectively 665 and 864 orthologues in *P. anserina*. We compared expression of *N. crassa* light regulated genes to their *P. anserina* orthologues in presence of bacteria, or during the vegetative incompatibility reaction.

|  | Up-regulated by blue light | | Down-regulated by blue light | |
| --- | --- | --- | --- | --- |
|  | Number of genes | E (p) | Number of genes | E (p) |
| Up VsSf | 272 | 2.35 (3e-17) | 78 | 0.5 (5e-7) |
| Up VsSm | 247 | 2.35 (1.9e-15) | 69 | 0.5 (8.5e-6) |
| Up inVI | 157 | 1.3 (ns) | 41 | 0.3 (7e-17) |
| Down VsSf | 69 | 0.6 (3.9e-4) | 421 | 2.75 (0) |
| Down VsSm | 53 | 0.5 (3.2e-4) | 390 | 3 (0) |
| Down in VI | 102 | 0.85 (ns) | 432 | 2.8 (0) |

E=Enrichment compared to expected, p=Two tails Fisher’s test

In a separate study, Chen and co-authors (EMBO J., 28(8), p1029-42, 2009) identified 288 genes up-regulated by blues light in *N. crassa*, 255 of which have orthologues in *P. anserina*. Based on their expression profiles they characterized the genes as Early Light Responsive (ELR) or Late Light Responsive (LLR) genes. ELR and LLR *N. crassa* genes have respectively 119 and 136 orthologues in *P. anserina*. We characterized the expression of ELR and LLR *P. anserina* orthologues in response to bacteria or during vegetative incompatibility.

|  | All Light Responsive | | ERL | | LLR | |
| --- | --- | --- | --- | --- | --- | --- |
|  | Number of genes | E (p) | Number of genes | E (p) | Number of genes | E (p) |
| Up VsSf | 133 | 2.7 (1e-14) | 43 | 2 (2e-3) | 84 | 3.5 (8.3e-14) |
| Up VsSm | 120 | 2.7 (7.6e-13) | 35 | 1.8 (9.9e-3) | 81 | 3.7 (1.3e-13) |
| Up inVI | 56 | 1.1 (ns) | 21 | 1 (ns) | 30 | 1.2 (ns) |
| Down VsSf | 24 | 0.5 (8.3e-4) | 14 | 0.6 (ns) | 3 | 0.1 (1e-5) |
| Down VsSm | 22 | 0.5 (5.8e-4) | 14 | 0.7(ns) | 2 | 0.1 (3.3e-5) |
| Down in VI | 34 | 0.7 (ns) | 16 | 0.75 (ns) | 14 | 0.6 (ns) |

E=Enrichment compared to expected, p=Two tails Fisher’s test

**Supplementary figure 10C:** Comparison of *P. anserina* response to non self to *N. crassa* response to non self.

Focusing on orthologous gene pairs we compared genes expressed in response to non self in *P. anserina* to genes differentially expressed during VI in *N. crassa* (Hutchison E, Brown S, Tian C, Glass NL.Microbiology. 2009 Dec;155(Pt 12):3957-70. doi: 10.1099/mic.0.032284-0).

|  |  |  | In *P. anserina* | | |
| --- | --- | --- | --- | --- | --- |
|  | Orthologous gene pairs | Up in Nc VI (844)  Nb / percent / pvalue | Up VsSf  Nb/ % / pvalue | Up VsSm  Nb/ % / pvalue | Up in VI  Nb/ % / pvalue |
| Up VsSF | 1164 | 149 / 12.8 / ns | - | 908 /78.1 / 0 | 428 / 36.7 / 0 |
| Up VsSm | 1023 | 129 / 12.6 / ns | 976 / 88.6 / 0 | - | 462/38.4/ 0 |
| Up in VI | 999 | 180 / 18 / 2.2^e-4^ | 427 / 42.8 / 0 | 393 / 39.4 / 0 | - |
|  |  | Down in Nc VI (1000)  Nb / percent / pvalue |  | | |
| Down VsSF | 1477 | 381 / 25.8 / 3.6^e-5^ | - | 1200 / 81.3 / 0 | 988 / 67 / 0 |
| Down VsSm | 1321 | 343 / 26 / 5.3^e-5^ | 1201 / 90.8 / 0 | - | 904 / 68.5 / 0 |
| Down in VI | 1634 | 447 / 27.3 / 6.1^e-8^ | 988 / 60.5 / 0 | 904 / 54.4 / 0 | - |
