# Supplementary figures and images for: Overlapping Podospora anserina Transcriptional Responses to Bacterial and Fungal Non Self Indicate a Multilayered Innate Immune Response
Source: Front Microbiol. 2016 Apr 19;7:471. doi: 10.3389/fmicb.2016.00471 (PMC4835503; doi:10.3389/fmicb.2016.00471)

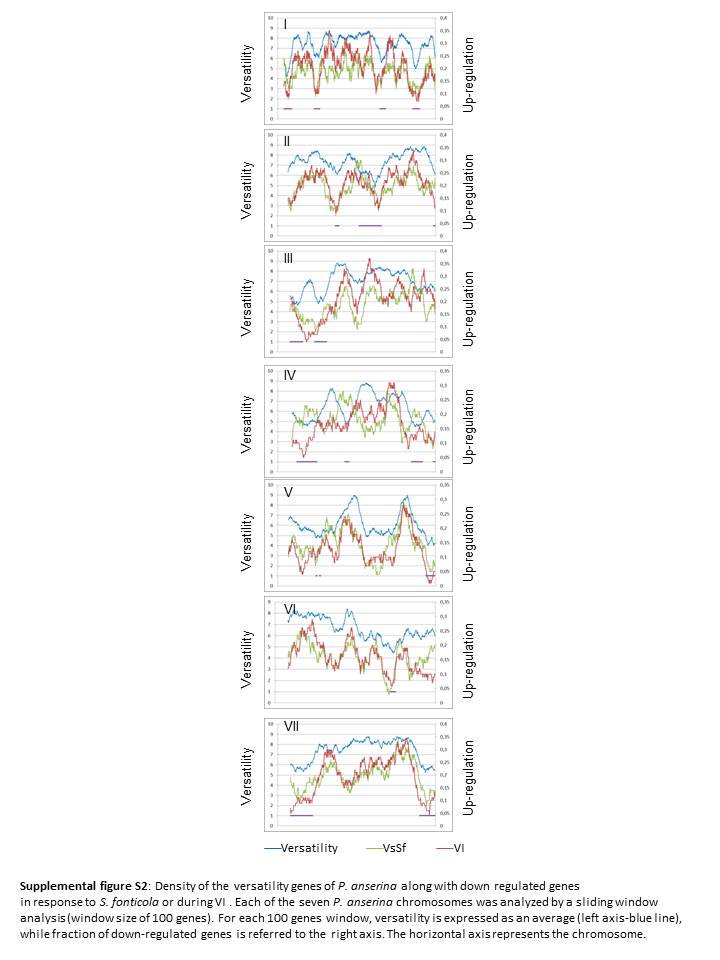

Supplement: Supplementary file 9 [file Image1.JPEG]

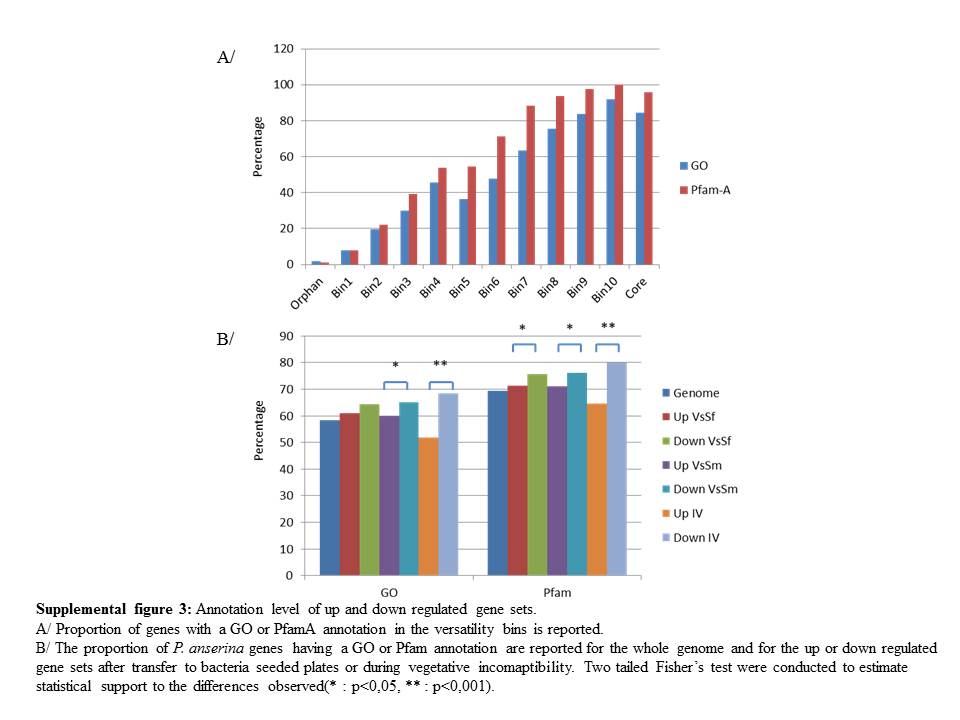

Supplement: Supplementary file 10 [file Image2.JPEG]
